# Evidence of Oxidative Stress as a Mechanism of Pharmaceutical-Induced Toxicity in Amphibians

**DOI:** 10.3390/antiox13111399

**Published:** 2024-11-15

**Authors:** Jesús Daniel Cardoso-Vera, Hariz Islas-Flores, Itzayana Pérez-Alvarez, Nidya Díaz-Camal

**Affiliations:** Laboratorio de Toxicología Ambiental, Facultad de Química, Universidad Autónoma del Estado de México. Paseo Colón Intersección Paseo Tollocan, Colonia Residencial Colón, Toluca 50120, Estado de México, Mexico; ipereza006@profesor.uaemex.mx (I.P.-A.); nidyadc@gmail.com (N.D.-C.)

**Keywords:** biomarkers, bioindicators, pharmaceuticals, redox imbalance, xenobiotics

## Abstract

Amphibians, which are essential components of ecosystems, are susceptible to pharmaceutical contamination, a phenomenon of increasing concern owing to the widespread consumption and detection of pharmaceutical compounds in environmental matrices. This review investigates oxidative stress (OS) as the primary mechanism of drug toxicity in these organisms. The evidence gathered reveals that various pharmaceuticals, from antibiotics to anesthetics, induce OS by altering biomarkers of oxidative damage and antioxidant defense. These findings underscore the deleterious effects of pharmaceuticals on amphibian health and development and emphasize the necessity of incorporating OS biomarkers into ecotoxicological risk assessments. Although further studies on diverse amphibian species, drug mixtures, and field studies are required, OS biomarkers offer valuable tools for identifying sublethal risks. Furthermore, the development of more refined OS biomarkers will facilitate the early detection of adverse effects, which are crucial for protecting amphibians and their ecosystems. Ultimately, this review calls for continued research and mitigation strategies to safeguard biodiversity from pharmaceutical contamination.

## 1. Introduction

Environmental pollution has become a critical global issue due to the release of various anthropogenic contaminants, including pharmaceuticals [1]. These compounds, designed for the prevention and treatment of diseases in humans and animals, are continuously introduced into the environment via various pathways, including wastewater discharge, improper pharmaceutical disposal, and agricultural runoff. The presence of pharmaceuticals in aquatic ecosystems is a matter of significant concern because of their persistence, potential for bioaccumulation, and possible adverse effects on non-target organisms [2]. Amphibians exhibit susceptibility to aquatic contaminants, including pharmaceuticals, owing to their permeable integument and biphasic life cycle. Exposure to these compounds can elicit deleterious effects in amphibians, encompassing alterations in developmental processes and reproductive functions as well as inducing damage at the cellular and molecular levels [3]. Oxidative stress, characterized by an imbalance between the production of reactive oxygen species (ROS) and the antioxidant capacity of cells, has been identified as a crucial mechanism of pharmaceutical-induced toxicity in diverse organisms, including amphibians [4]. Consequently, elucidating the effects of pharmaceutical compounds on amphibian populations is crucial not only for the preservation of biodiversity, but also for evaluating potential risks to human health, given the intricate interconnections between aquatic ecosystems and human well-being.

### 1.1. Amphibians

The taxonomic category Amphibia comprises 72 families and 562 genera, encompassing over 8350 species. Approximately 3% of the total are caecilians, 9% are caudates, and 88% are frogs [5]. Most amphibians exhibit a biphasic life cycle characterized by the presence of both aquatic and terrestrial stages. The larval phase, which is typically aquatic, transitions to the adult phase, which can reside in either water or land environments. During their development into adults, larvae undergo metamorphosis, which involves the transition from using gills to breathing air. Amphibians typically rely on moist skin as a secondary respiratory surface [5].

The Gymnophiona, Caudata, and Anura clades contain all the amphibian species [6]. Caecilians are legless burrowing amphibians belonging to the order Gymnophiona, which inhabit humid tropical areas in the Americas, Asia, and Africa. Most caecilians are less than 50 cm in length and are markedly similar to earthworms. They have small, highly ossified heads with annular grooves along the body, and degenerated eyes [7]. Caecilians are a diverse group of organisms, with approximately 200 different species identified to date. Despite their elusive nature, much of their biology remains unclear [8,9].

Salamanders belong to the order Caudata, which consists of over 700 species and is categorized into three groups: sirens (eel-like amphibians), basal salamanders (including hellbenders), and derived salamanders (mudpuppies, amphiumas, axolotls, newts, and various terrestrial species) [7,10]. Although it is widely believed that salamanders begin their lives as larvae in water and eventually transform into adult forms that live on land, this notion is not accurate for most salamander species [11]. Specifically, two-thirds of salamander species belong to the lungless salamander family (Plethodontidae), and these animals hatch directly from their eggs into terrestrial forms. Moreover, certain species, such as *Necturus maculosus* (commonly known as mudpuppy) and *Ambystoma mexicanum* (axolotl), exhibit neoteny, the retention of juvenile characteristics into adulthood. In these species, individuals can reach sexual maturity while still in their larval form and may never undergo metamorphosis [6].

The order Anura, which comprises toads and frogs, is the amphibian order with the greatest number of extant species, totaling 7708 [12]. These animals are distinguished by their small, tail-free bodies, damp and porous skin, large eyes, and long and powerful hind legs, which enable them to jump effectively. In most cases, their life cycle includes an aquatic larval stage with gills that transforms into a terrestrial adult with lungs. Anurans are found in a diverse range of ecosystems, encompassing tropical forests and deserts, and fulfill a vital function as both predators and prey for other species [7,8]. The significance of this order as an essential subject of study arises from its taxonomic and functional diversity, which enables researchers to understand the evolution and adaptation of vertebrates to various ecosystems. The order Anura comprises 55 families that are currently recognized and can be categorized into 11 superfamilies: Hyloidea, Dendrobatoidea, Microhyloidea, Ranoidea, Pelobatoidea, Pipoidea, Rhinophrynoidea, Scaphiopodoidea, Discoglossoidea, Pelodytoidea, and Leiopelmatoidea [12,13,14]. The ongoing classification of amphibians is subject to change as new phylogenetic research emerges, and additional species are uncovered.

### 1.2. Emerging Contaminants

The origin of emerging contaminants (ECs) can be traced back to the Industrial Revolution, which introduced a plethora of new chemicals into the environment. Although the concept of these substances as potential environmental contaminants has recently gained traction, it was not a major concern during the earlier periods. Throughout the Industrial Revolution, until the late 20th century, conventional pollutants were the primary focus, with the recognition and comprehension of ECs undergoing substantial development in the latter half of the 20th century. The evolution of analytical techniques in the early 21st century has played a crucial role in the detection of low concentrations of new chemicals, leading to a significant shift in the perception of ECs [15].

Emerging contaminants, which are substances that possess characteristics such as biotoxicity, environmental persistence, and bioaccumulation, are released into the environment and pose risks to both the ecological environment and human health. Despite these risks, their regulations and management have not been comprehensively addressed or effectively implemented [16].

Depending on their chemical characteristics, use, and origin, ECs can be classified into several categories: (I) pharmaceuticals and personal care products (PPCPs). This group of substances, intended to enhance health, hygiene, and aesthetics, is of increasing concern because of their environmental presence. It includes various compounds, such as prescription and over-the-counter medications, as well as personal care and grooming products, such as lotions, cosmetics, and fragrances [15]. (II) Endocrine-disrupting chemicals (EDCs). Endocrine-disrupting compounds (EDCs) can impair the normal function of glands and hormones by blocking, mimicking, or disrupting their natural actions. Consequently, EDCs may adversely affect vital functions such as growth, development, and reproduction [17]. Certain pharmaceuticals compounds may function as endocrine disruptors and potentially exert significant effects on ecosystem health [18,19]. (III) Polyfluoroalkyl substances (PFASs). These compounds possess numerous carbon–fluorine bonds, imparting unique properties, such as resistance to heat, water, and oil. Their chemical and thermal stabilities have facilitated their use in diverse products, including firefighting foams, waterproof coatings, nonstick cookware, and various manufacturing processes [20]. (IV) Microplastics (MPs). These tiny plastic fragments, less than 5 mm in length, include primary PMs, originally manufactured at this size, and secondary PMs, which result from the degradation of larger macroplastics (>5 mm) under various environmental conditions. The widespread presence of microplastics in the air, soil, water, and organisms significantly threatens human health and ecosystem balance [21]. (V) Nanomaterials. Materials sized between 1 and 100 nm exhibit unique physical, chemical, and biological properties owing to their nanometer scale. This category comprises carbon nanotubes, graphene, quantum dots, metal nanoparticles, and ceramic nanofibers. Nonetheless, their small size and high surface-to-volume ratio raise concerns about their potential adverse effects on human health and the environment [15]. (VI) Industrial chemicals and by-products (ICBs). This category encompasses manufacturing chemicals and persistent environmental toxins such as dioxins. Additionally, heavy metals, such as lead and mercury, can contaminate soil and water, harming ecosystems and human health. The improper disposal of organic solvents and pesticides can disrupt natural cycles and the food chain [22,23].

### 1.3. Pharmaceuticals in the Environment

Pharmaceutically active compounds (PhACs), natural or synthetic chemical compounds with specific biological activities, are mainly used to prevent, diagnose, treat, or alleviate disease symptoms [24]. The traditional definition of “drug” suggests that most of these compounds act in a similar way in any biological system, depending on their mechanism of action. However, it is important to recognize that amplified or unexpected effects may arise in certain species owing to physiological variations, including differences in the manner in which the drug is metabolized and interacts with the organism [25].

PhACs enter the environment primarily through two pathways, both through their use in humans and animals and their subsequent metabolic excretion in urine and feces (including unmetabolized parent drugs, drug conjugates, and bioactive metabolites), and the improper disposal of leftover or expired medications in the sewage system [26]. PhACs that undergo incomplete degradation in wastewater treatment plants (WTPs) are released into treated effluents, causing the detection of these compounds in surface water, seawater, groundwater, and even drinking water at concentrations ranging from ngL^−1^ to μgL^−1^ [27,28,29,30,31,32,33,34,35,36,37]. In addition, irrigation with treated effluents or the application of sludge from WTPs (biosolids) in crop fields is a common pathway for soil contamination with PhACs [38,39,40].

PhACs are present at low concentrations in the environment, typically ranging from ngL^−1^ to µgL^−1^, but their pervasiveness poses a potential threat to organisms in both aquatic and terrestrial ecosystems [41,42,43]. The persistent nature of several PhACs increases the possibility of bioaccumulation in different organisms, which can lead to serious physiological disorders [44,45,46]. Although some other PhACs do not present this persistence characteristic, through their continuous release and constant presence in the environment, they exhibit a “pseudopersistent” behavior, representing a risk to living organisms [47,48].

Wildlife can play a key role in assessing environmental contamination by PhACs, acting as sentinels, monitoring bioaccumulation, and serving as a bioindicator of potential adverse effects depending on the environmental fate and mechanism of action of each PhAC. In addition, we must recognize the importance of the interconnection between human, animal, and environmental health, highlighting the need to further investigate the impact of PhACs on wildlife, especially considering their role in complex food webs involving humans [24].

### 1.4. Oxidative Stress

Oxidative stress (OS) is characterized by an imbalance between oxidative and antioxidant species, favoring the former. This imbalance disturbs signaling and redox control and can lead to damage at the molecular level [49]. A healthy redox system is based on the balance between oxidation and reduction, which implies a net-zero electron flow at the end of the biological pathways. Alterations in this redox steady state trigger molecular changes that, depending on their magnitude, can result in damage at the molecular, cellular, and/or tissue levels. Both endogenous and exogenous sources lead to constant OS exposure [50].

Reactive oxygen species (ROS) and reactive nitrogen species (RNS) are unstable molecules generated during aerobic cellular metabolism. Although they play crucial roles in cell signaling and immune responses, their excessive production can lead to oxidative damage of biomolecules such as lipids, proteins, and DNA [51,52]. Table 1 summarizes the major ROS and RNS, their formation, and the neutralization or elimination mechanisms in aerobic organisms.

Various environmental pollutants, including pharmaceuticals, can increase ROS production in amphibians and other aquatic organisms, inducing oxidative stress. Xenobiotics can elevate intracellular levels of ROS through various mechanisms, such as increased basal metabolism with the consequent intensification of mitochondrial activity, alteration of the redox cycle, increased generation of ROS as by-products of reactions mediated by cytochrome P450 enzymes (CYPs), or increased Fenton and Haber–Weiss reactions due to excess copper and iron ions. Oxidative stress biomarkers, which are first-line responses in animals and are highly sensitive to pollutants even at low concentrations, are useful for the early detection of environmental contamination [4].

#### 1.4.1. Oxidative Stress Biomarkers

##### Oxidative Damage

**Figure 1:**
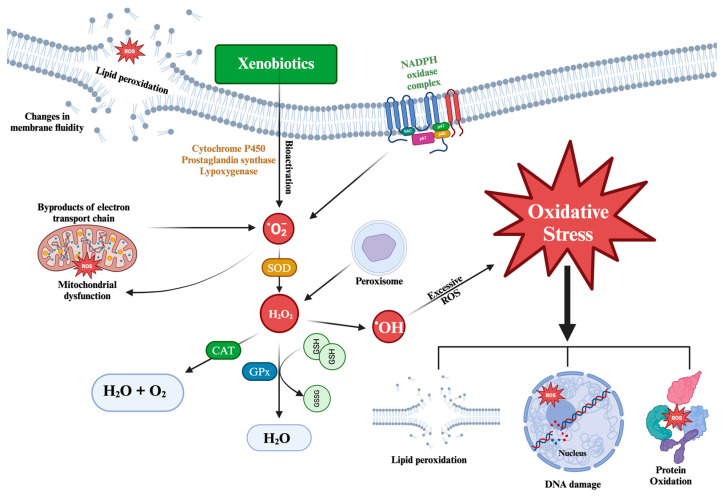
Mechanism and main consequences of oxidative stress at the cellular level.

Excessive ROS can damage various biomolecules including DNA, lipids, proteins, and carbohydrates (Figure 1). This damage can be detected using OS-specific biomarkers, which identify the molecular fingerprints left by ROS [53].

Lipids: When the concentration of ROS inside the cell increases, lipid peroxidation (LPO) is triggered. This process is initiated by the attack of a free radical on the methylene group of the fatty acid to extract a hydrogen atom. Consequently, a carbon-centered lipid radical (L•) is formed, which generates a lipid peroxide radical (LOO•) upon reaction with molecular oxygen. Subsequently, lipid peroxide undergoes a cyclization reaction to form an endoperoxide, which eventually decomposes into toxic end products such as malondialdehyde (MDA), 4-oxy-2-nonenal (ONE), and 4-hydroxyl nonenal (4-HNE) [50]. These end products are detrimental to the cell as they can cause damage to proteins and DNA. In addition, lipid peroxidation compromises the functionality of the cell membrane, decreasing its fluidity and inactivating cell membrane-anchored receptors and enzymes [54].

Proteins: Protein oxidation involves the participation of various ROS and propagating radicals. These reactions lead to oxidative modifications in amino acid side chains, ROS-mediated peptide fragmentation, reactions between peptides and lipids or carbohydrate oxidation products, and the formation of carbonyl derivatives of proteins [50]. The carbonylation of protein residues has been established as a key biomarker to assess protein damage caused by ROS, affecting specific amino acid residues, such as lysine, threonine, proline, and arginine [55,56].

DNA and RNA: Free radicals and ROS can induce specific modifications and hydroxylation in the purine and pyrimidine bases of DNA, damage the deoxyribose–phosphate backbone, and disrupt protein–DNA cross-linkage formation [54]. Mitochondrial DNA is more vulnerable to this type of damage than nuclear DNA due to its closer proximity to the site of ROS generation. In addition, there is scientific evidence that RNA is more susceptible to oxidative damage than DNA, due to factors such as its proximity to mitochondria (the main source of ROS), its single-stranded structure, the lack of an active repair mechanism for oxidative damage, and lower protection by proteins compared to DNA [50]. One of the most investigated biomarkers of damage is 7,8-dihydro-8-deoxyguanosine (8-oxoG) [57,58].

##### Antioxidant Defenses

The constant exposure to oxidizing factors, both internal and external, has led aerobic organisms to develop complex antioxidant mechanisms, including enzymes and other molecules, to maintain the redox balance. The study of these antioxidant enzymes has made it possible to assess an organism’s ability to cope with OS and, therefore, can be used as a biomarker [59].

The enzymes superoxide dismutase (SOD), catalase (CAT), and glutathione peroxidase (GPx) constitute the first line of cellular antioxidant defense. These enzymes are functionally interrelated because hydrogen peroxide (H_2_O_2_), the product of the reaction catalyzed by SOD, is the substrate for both CAT and GPx [60].

SOD is a crucial metalloenzyme involved in cellular defense against ROS. It acts as the first line of detoxification and is the most potent antioxidant within cells [61]. Its main function is to catalyze the conversion of two highly reactive superoxide anions (•O_2_^−^) into hydrogen peroxide (H_2_O_2_) and molecular oxygen (O_2_), thus reducing its damaging potential. SOD activity depends on the presence of a specific metal cofactor, resulting in different enzyme forms depending on the type of metal ion required [62].

CAT, which is widely distributed in living tissues that utilize oxygen, uses iron or manganese as a cofactor. Its main function is to catalyze the decomposition of hydrogen peroxide (H_2_O_2_) into water and O_2_, thus completing the detoxification process initiated by SOD [63].

GPx is an important intracellular enzyme that breaks down H_2_O_2_ into water and lipid peroxides into their corresponding alcohols, mainly in the mitochondria and occasionally in the cytosol. In most cases, its activity depends on selenium (Se) as a cofactor. Therefore, GPx is often referred to as selenocysteine peroxidase. This enzyme plays a crucial role in inhibiting the lipid peroxidation process, thus protecting OS cells [61,64].

Amphibians, owing to their sensitivity to chemical contaminants during their life cycles in water, are considered excellent bioindicators of environmental pollution. The decline in their populations in recent decades has been attributed to the combined effects of pollution, changes in human activity, and climate [65,66]. Despite their importance as indicators of environmental health, toxicological research on amphibians has been limited compared to that on other vertebrates [67,68]. The current study seeks to provide a comprehensive and critical analysis of the scientific literature to evaluate the existing evidence on the use of oxidative stress biomarkers as indicators of toxicity caused by various pharmaceutical compounds in different species of amphibians. This review aims to be exhaustive in its scope and provide a critical examination of the available evidence on this topic. The objective of analyzing these biomarkers is to identify response patterns, assess the sensitivity of various species, and determine the relevance of these biomarkers in conducting ecotoxicological risk assessments.

## 2. Methodology

An exhaustive bibliographic search was carried out in the electronic databases Web of Science, PubMed, and Scopus, covering the period 2000 to 2024. Combinations of the following search terms were used: oxidative stress, amphibians, drugs, pharmaceuticals, biomarkers, oxidative damage, SOD, CAT, and GPx.

The inclusion criteria were as follows:

- Experimental investigations analyzing the impact of drug exposure on oxidative stress biomarkers in amphibians.
- Articles published in peer-reviewed scientific journals in English.
- Studies providing data on at least one oxidative stress biomarker and drug-specific class.
- Research that clearly identifies the amphibian species used in this study.

The exclusion criteria were as follows:

- Studies using plant extracts or natural compounds instead of synthetic drugs.
- Studies that did not provide quantitative data on biomarkers of oxidative stress.
- Studies that focus exclusively on drug bioaccumulation without evaluating biomarkers of oxidative stress.
- Studies that do not provide sufficient information on experimental conditions.

Data extraction was performed systematically, documenting the following information for each study: authors, year of publication, amphibian species used, type of drug, concentration used, exposure time, main results, and conclusions.

## 3. Results

This review identified several studies that have investigated the effects of various pharmaceuticals on biomarkers of oxidative stress in amphibians. The results revealed complex and varied responses depending on both the type of drug and amphibian species studied (Table 2). It is important to note that all studies to date have focused on frog and toad species, such as *Rhinella arenarum*, *Xenopus laevis*, and *Lithobates catesbeianus*. However, amphibian groups of salamanders and caecilians remain largely unexplored in this context, representing a substantial gap in our understanding of the impact of pharmaceuticals on amphibian biodiversity.

### 3.1. Antibiotics

Antibiotics are pharmaceutical agents extensively utilized in both human and veterinary medicine to combat infectious diseases. Furthermore, they are administered at subtherapeutic doses as feed additives in animal husbandry to promote growth, a practice that has been prevalent in animal husbandry, but is presently being regulated or prohibited in numerous regions due to concerns regarding antimicrobial resistance [85]. The global consumption of antibiotics is estimated to range between 100,000 and 200,000 metric tons. Nevertheless, a substantial proportion, ranging from 70% to 90%, is not fully metabolized within an organism and is subsequently excreted in its original form or as active metabolites, thereby entering the environment [86]. Previous studies have provided information on the toxicity of antibiotics to algae [87,88], microcrustaceans [89,90,91], mollusk bivalves [92,93,94], and fish [95,96,97,98]. Nevertheless, research on the impact of antibiotics on amphibian populations remains limited, despite their importance in assessing environmental risk as bioindicators of potential environmental contamination in the context of ecosystem imbalance. The lethal and sublethal effects of oxytetracycline (OTC) were evaluated in *Rhinella arenarum* embryos exposed to 10–115 mgL^−1^ for a period of 96 h. These findings indicated that OTC exposure significantly altered the activity of antioxidant enzymes, including decreased CAT, SOD, and GST activity. In addition, an increase in GSH levels was observed. Although no oxidative damage was evident, the authors suggested that continued exposure to OTC might have long-term negative effects [78]. In another study, da Luz et al. (2021) [76] utilized *Physalaemus cuvieri* tadpoles to assess the acute toxicity of azithromycin (AZT) and hydroxychloroquine (HCQ). Tadpoles were exposed to a concentration of 12.5 μgL^−1^ of the drugs for 72 h. This concentration was selected to simulate the potential increase in the environmental drug concentrations attributable to the COVID-19 pandemic. The study found no evidence of increased oxidative damage in tadpoles exposed to the drug, as assessed by biomarkers, such as nitrite, TBARS, ROS, and H_2_O_2_. However, an increase in SOD and CAT activities was observed, suggesting an adaptive response to counteract potential oxidative stress. In addition, the toxicity of two veterinary antibiotics, enrofloxacin (ENR, veterinary medicine) and ciprofloxacin (CPX, human medicine), was evaluated in *R. arenarum* larvae exposed to environmentally relevant concentrations of both drugs (1–1000 μgL^−1^) for 96 h under standard laboratory conditions. Concentrations higher than 10 μgL^−1^ of both antibiotics induced detrimental effects on larvae, mainly on development, growth, and antioxidant enzyme activity. Specifically, CPX at 1000 μgL^−1^ induced a significant increase in GST activity and ENR at 1000 μgL^−1^ inhibited both GST and CAT. These findings suggest that the tested antibiotics can trigger EO and affect antioxidant defense mechanisms in *R. arenarum* larvae [72]. The effects of two commonly used antibiotics, sulfamethoxazole (SMX) and oxytetracycline (OTC), were evaluated in tadpoles of *Lithobates catesbeianus*, which were exposed to 20, 90 and 460 ngL^−1^ of both antibiotics for 16 days. OTC, especially at the highest concentrations, led to a decrease in SOD, GPx, and glucose 6-phosphate dehydrogenase (G6PDH) activities. In addition, increased levels of carbonylated proteins were observed in the liver of tadpoles exposed to the highest concentration of OTC. In contrast, SMX did not significantly affect the evaluated biomarkers [79]. Finally, one study evaluated the toxicity of a commercial formulation of monensin (CFM), a polyether antibiotic isolated from *Streptomyces cinnamonensis*, on embryos and larvae of *R. arenarum* exposed to 4, 12, and 20 μgL^−1^ following acute exposure for 96 h. In embryos, a significant decrease in GST activity was observed at 12 and 20 μgL^−1^. In larvae, GST activity was significantly decreased at 20 μgL^−1^. In addition, low GSH levels were observed in larvae exposed to 12 and 20 μgL^−1^, and an increase in lipid peroxidation was observed at all concentrations tested [83].

### 3.2. Nonsteroidal Anti-Inflammatory Drugs

Nonsteroidal anti-inflammatory drugs (NSAIDs) are a group of drugs with analgesic, anti-inflammatory, and antipyretic properties. Their widespread use worldwide, with billions of prescriptions dispensed each year, makes them one of the most widely consumed types of drugs [99]. In addition, NSAIDs have been shown to be resistant to degradation, persistent, pharmacologically active, and toxic to non-target organisms such as algae [100,101], molluscs [102,103], and fish [104,105,106]. As for the studies in amphibians, embryos and larvae of *Trachycephalus typhonius* and *Physalaemus albonotatus* were exposed to concentrations between 125 and 2000 μgL^−1^ for 22 days. In *T. typhonius*, GST activity was inhibited at low concentrations (125 μgL^−1^) but induced at high concentrations (500, 1000 and 2000 μgL^−1^). In *P. albonotatus*, GST activity was inhibited at both low and high concentrations, exhibiting a hormesis-type response pattern, with maximal activity at intermediate concentrations. GST activity shows complex responses and varies by species, highlighting the importance of considering species-specific sensitivity when assessing the ecological risk of these compounds [73]. In another study, adult male *Pelophylax ridibundus* frogs were exposed to 250 ngL^−1^ ibuprofen for 14 days. These findings indicate that ibuprofen exposure caused an increase in oxyradicals and glutathione levels (both reduced and oxidized); however, SOD activity was not affected. Additionally, the concentration of lipofuscin, a marker of oxidative damage, decreased with drug exposure.

### 3.3. Antivirals

Antivirals (AVs), drugs designed to combat viral infections by limiting or preventing viral replication, have increased in consumption. Consequently, an increase in the concentrations of these compounds and their active metabolites in the environment has been observed [107]. The presence of AVs in ecosystems pose a potential risk, because they can interfere with the normal functions of biological systems [108]. *Rhinella arenarum* tadpoles were exposed to four antiretrovirals (lamivudine (3TC), stavudine (d4T), zidovudine (AZT), and nevirapine (NVP)) at concentrations ranging from 0.5 to 4 mgL^−1^ for a period of 48 h to determine the acute toxicity of these compounds. Among the four compounds, 3TC showed the lowest bioaccumulation. A statistically significant increase in GST activity was observed only at the highest concentration tested. The bioaccumulation of d4T was slightly higher than that of 3TC. In addition, the increase in GST activity was statistically significant at 1, 2, and 4 mgL^−1^. AZT showed low bioaccumulation in tadpoles. GST activity similarly increased at concentrations of 1, 2, and 4 mgL^−1^. Finally, NVP exhibited the highest bioaccumulation of all tested compounds, suggesting a high permeability to tadpoles. The increase in GST activity was statistically significant at all the four concentrations tested. The observed increase in GST activity indicates the overproduction of ROS, which can cause oxidative damage in *R. arenarum* tadpoles [75]. A recent study evaluated the toxicity of two VA, favipiravir (32.9–250 mgL^−1^) and oseltamivir (8.2–62.5 mgL^−1^), in *Xenopus laevis* embryos and tadpoles for a 96 h period. Favipiravir inhibits the activity of GR and CAT enzymes in embryos; however, it increases GST activity and decreases MDA levels. In embryos exposed to oseltamivir, no significant alterations in CAT activity or MDA levels were observed. In tadpoles, both GST and GR activities and MDA levels decreased. The results suggest that VA may affect antioxidant defense systems in *X. laevis*, and that the response varies depending on the developmental stage of the organism [81].

### 3.4. Antihypertensive

The recurrent detection of antihypertensive drugs in the environment reflects the accelerated growth of the pharmaceutical industry and the high consumption of these drugs globally [109]. Additionally, the presence of these drugs in the environment can have harmful effects on organisms, affecting their health and survival [110,111,112]. A study was conducted on adult male frogs of the species *Pelophylax ridibundus* to evaluate the toxicity of nifedipine at a concentration of 10 μM for a period of 14 days. Drug exposure causes oxidative stress, as evidenced by a considerable increase in the rate of ROS generation and SOD, GSH, and GSSG levels [70].

### 3.5. Glucocorticoids

Glucocorticoids (GC) are widely used in the treatment of various diseases, such as rheumatoid arthritis, asthma, and Crohn’s disease, owing to their potent anti-inflammatory properties [113]. Both natural and synthetic GCs exert their main action through the glucocorticoid receptor (GR), which modulates the expression of specific genes. In addition, they can act through non-genomic mechanisms, binding directly to the GR without affecting gene expression [114]. The increase in the human population, together with its aging, has led to a greater dependence on GCs, as is the case with other drugs. This growing demand raises concerns regarding the potential environmental impacts of the consumption and presence of these drugs in the environment [115]. Among the studies that have evaluated the toxicity of glucocorticoids in amphibians, Cuzziol Boccioni et al. (2020) [74] evaluated the chronic toxicity of dexamethasone at concentrations of 1–1000 μgL^−1^ in *Rhinella arenarum* larvae for an exposure period of 22 days. The results showed a significant increase in GST activity in the larvae exposed to dexamethasone, indicating a response to oxidative stress. In addition, the authors related the histological alterations observed in different tissues, such as intestinal dysplasia and epithelial cells, to oxidative damage induced by the drug. Rutkoski et al. (2024) [84] evaluated the toxicity of two glucocorticoids, prednisone (PD) and prednisolone (PL), on *Aquarana catesbeianus* tadpoles exposed to concentrations of 0.1, 1, and 10 μgL^−1^ for 16 days. PD exposure caused an increase in the MDA levels. In addition, both drugs caused an increase in CAT and GPx activities, while PD exposure also elevated SOD, GST, and G6PDH activities. These findings suggest that GCs induce oxidative stress in tadpoles of *A. catesbeianus*.

### 3.6. Pharmaceutical Mixture

Research has demonstrated that individual pharmaceutical compounds can influence diverse molecular and cellular pathways in various non-target organisms. Furthermore, the nature and intensity of the effects were directly correlated with the dosage and specific compound. However, it is crucial to consider that under real conditions, organisms are often exposed to low doses of multiple drugs simultaneously. This combined exposure can modulate the overall toxicity through different pathways; however, they often interact with each other, adding to the complexity of assessing the environmental impact of pharmaceuticals [116]. A study was conducted to evaluate the effect of a pharmaceutical mixture comprising diclofenac, naproxen, atenolol, and gemfibrozil on *Limnodynastes peronii* tadpoles. The subjects were exposed to various concentrations of the mixture (0.1, 1, 10, 100, and 1000 μgL^−1^) for a 30-day period. The results showed that peroxidase activity increased significantly in tadpoles exposed to a 1000 μgL^−1^ concentration, suggesting an increase in oxidative stress. However, no significant effects on SOD or RBC activity were observed at any of the tested concentrations [69].

### 3.7. Anesthetic

Tricaine methanesulfonate (MS-222) is the anesthetic of choice for amphibians and fish, with applications ranging from simple procedures requiring sedation, such as morphometric measurements, to its use as part of euthanasia protocols, either as a first step or as the sole agent [117,118]. A study conducted by Gavrilović et al. (2024) [80] investigated the potential impact of MS-22 as an anesthetic/euthanizing agent on experimental outcomes in studies examining biomarkers of oxidative stress. *Hyla arborea* tadpoles were reared at two different temperatures (20 °C and 25 °C) to induce variations in their antioxidant capacities. Subsequently, the tadpoles were exposed to 0.1, 1, and 5 gL^−1^ of MS-222 for 15 min. The results of this study indicated that MS-222 can significantly alter GSH levels and GSH/thiol-related parameters. Furthermore, specimens from different temperature groups exhibited varying responses to MS-222, suggesting a potential correlation with initial levels of antioxidant capacity. The biomarkers of oxidative damage and CAT activity were not significantly affected by MS-222 exposure.

### 3.8. Benzodiazepines

Benzodiazepines (BDZs), which are psychotropic drugs widely prescribed worldwide, are used to treat mental disorders, including anxiety, panic disorder, and insomnia. Its therapeutic action is due to its interaction with the γ-aminobutyric acid (GABA) receptor, which results in an increase in ionic conduction and the consequent manifestation of anxiolytic, hypnotic, and sedative effects [119,120]. The presence of BDZs in the environment poses a potential risk due to their ability to be absorbed by organisms. Several studies have demonstrated the ability of these compounds to bioaccumulate and cause adverse effects in various aquatic organisms [121,122,123,124]. In amphibians, limited research has been conducted on the effects of BDZs on these organisms. Fogliano et al. (2022) [77] exposed *X. laevis* embryos to 1, 5, and 10 μgL^−1^ of delorazepam (DLZ) from the 4–8 cell stage to stage 45–46, simulating early and prolonged exposure to the drug in a natural environment. DLZ exposure caused a significant increase in ROS production and lipid hydroperoxide levels, indicating oxidative damage at the cellular level. In response to stress, an increase in GPx and GR activity was detected, although this response was not sufficient to completely counteract the oxidative damage induced by the drug, especially at the highest concentration.

### 3.9. Antiparasitic

The increasing use of macrocyclic lactones for the control of parasitic infections has been accompanied by significant historical developments. The first macrocyclic lactones used to combat parasites (roundworms and arthropods) were avermectin and its chemical derivative ivermectin (IVM) [125]. IVM, owing to its broad-spectrum anthelmintic activities, is used in the treatment of various diseases caused by parasitic nematodes [126]. Previous studies have shown adverse effects of IVM on non-target organisms in both terrestrial and aquatic ecosystems [127,128,129,130,131]. Embryos and larvae of *Rhinella arenarum* were exposed to different concentrations of IVM, both the active ingredient and a commercial formulation, varying between 1.5, 5, and 10 μgL^−1^ for 96 h. Exposure to IVM, both in its active ingredient form and in a commercial formulation, induced significant changes in oxidative stress biomarkers. CAT activity was increased in embryos exposed to the commercial formulation and in larvae exposed to both forms of IVM. GST activity increased in embryos and larvae exposed to the commercial formulation but was inhibited in embryos exposed to the active ingredient. In addition, GSH levels were decreased in embryos exposed to the active ingredient. TBARS levels increased in embryos and larvae exposed to the commercial formulation. This study highlighted that the commercial formulation of IVM is more toxic than the active ingredient, underscoring the importance of considering the effects of excipients in environmental risk assessments [82].

## 4. Discussion

The findings of this review underscore the significance of incorporating oxidative stress considerations into the ecotoxicological risk assessments of pharmaceuticals. The use of oxidative stress biomarkers can yield valuable insights into the sublethal effects of pharmaceutical exposure and facilitate the identification of potential risks to amphibian populations [4].

Nevertheless, research in this field remains limited, and there is a substantial scope for further investigation. It is imperative to expand the species spectrum, as no studies have been conducted on salamanders (order Caudata) or caecilians (order Gymnophiona), which limits our understanding of the effects of pharmaceuticals on amphibian diversity [132]. Most of the reviewed studies have predominantly focused on the short-term effects of individual pharmaceutical compounds under controlled laboratory conditions, thereby limiting their extrapolation to real-world scenarios in which amphibians are exposed to multiple contaminants and environmental stressors [133]. Further research is necessary to investigate the effects of pharmaceutical combinations and to conduct field studies to adequately assess the impact of pharmaceuticals on natural ecosystems.

Furthermore, it is imperative to develop more sensitive and specific biomarkers for the early detection of adverse drug effects in amphibians. Therefore, antioxidant enzymes should be evaluated in conjunction with other biomarkers of oxidative damage for a more comprehensive assessment of oxidative stress [134].

It is imperative to note that ecotoxicological studies on amphibian species that investigate the chemical toxicity of environmental substances and pollutants remain limited. Historically, a greater emphasis has been placed on aquatic organisms such as insects, crustaceans, fish, and algae, while amphibians have received comparatively less attention [135]. Significant data gaps persist, including the absence of experimental data for various amphibian life stages and the lack of environmental legislation mandating the environmental risk assessment of chemicals, including pharmaceuticals, in amphibians [136].

An additional significant consideration is that despite the existence of guidelines, such as the ASTM-FETAX test and OECD Tests 231 and 241, which provide recommendations for assessing toxicity in amphibians, the absence of a standardized guideline encompassing all life stages and diverse toxicity endpoints impedes the comparison of results across studies and extrapolation of effects to wild populations. This underscores the necessity for a universally accepted guideline that promotes consistency in experimental designs and facilitates the environmental risk assessment of pharmaceutical across diverse experimental protocols and research objectives [136].

Amphibians are regarded as exemplary bioindicators of potential environmental contamination and should be utilized in monitoring and environmental risk assessment processes for pharmaceuticals [137,138]. Pharmaceuticals are subject to environmental risk assessments; however, current approaches frequently omit amphibians, raising significant concerns regarding their protection. Existing methodologies may be insufficient because amphibians exhibit sensitivity patterns that are different from those of other species [139].

Finally, it is imperative to continue investigating the effects of pharmaceuticals on amphibians and other aquatic organisms as well as to develop effective strategies to mitigate their adverse effects. Only through a comprehensive understanding of the risks associated with pharmaceutical contamination can we safeguard the integrity of ecosystems and ensure the preservation of the species inhabiting them.

## 5. Conclusions and Future Research

This review provides a comprehensive analysis of the role of oxidative stress in the mechanism underlying drug-induced toxicity in amphibians. Research has demonstrated that a diverse array of pharmaceutical compounds, including antibiotics, nonsteroidal anti-inflammatory drugs, antivirals, antihypertensives, glucocorticoids, anesthetics, and benzodiazepines can elicit oxidative stress in these organisms. Alterations observed in the biomarkers of oxidative damage include increased ROS production, lipid peroxidation, and protein oxidation, along with changes in the activity of antioxidant enzymes, highlighting the detrimental effects of these compounds on amphibian health and development. The results of this review emphasize the significance of considering oxidative stress in the ecotoxicological risk assessments of pharmaceuticals, a methodological process that aims to determine the probability of adverse effects occurring in the environment due to the presence of these compounds. This process encompasses the identification of hazards, evaluation of exposure, and characterization of risk. The use of oxidative stress biomarkers can provide valuable information regarding the sublethal effects of drug exposure and can facilitate the identification of potential risks to amphibian populations.

However, research in this field is still limited and there is room for improvement. It is crucial to expand the species spectrum as no studies have been performed on salamanders (order Caudata) or caecilians (order Gymnophiona), which limits our understanding of the effects of drugs on amphibian diversity. This review identified only one study that evaluated the effects of a drug mixture. Most of the reviewed studies have focused on the short-term effects of individual drugs under laboratory conditions, which limits their extrapolation to real situations where amphibians are exposed to multiple contaminants and stressors. Further research is required to investigate the effects of pharmaceutical combinations and conduct field studies to adequately assess the impact of pharmaceuticals on the natural environment. Additionally, the development of more sensitive and specific biomarkers is essential for the early detection of adverse drug effects in amphibians.

Finally, it is imperative to continue researching the effects of drugs on amphibians and other aquatic organisms as well as to develop effective strategies to mitigate their negative effects. Only through a comprehensive understanding of the risks associated with pharmaceutical pollution can we safeguard the health of ecosystems and ensure the preservation of the species inhabiting them.

## Figures and Tables

**Table 1:** Formation and neutralization/removal of major reactive oxygen and nitrogen species.

Reactive Species	Formation	Neutralization/Elimination
Reactive Oxygen Species (ROS)		
^.^O_2_^−^ Superoxide anion	It is primarily generated as a byproduct of the electron transport chain in the mitochondria, as well as by the activity of enzymes such as NADPH oxidases and xanthine oxidase.	Superoxide dismutase (SOD) catalyzes the dismutation of superoxide to hydrogen peroxide. Spontaneous dismutation may also occur.
^∙^OH Hydroxyl radical	Formed through the Fenton reaction, where hydrogen peroxide reacts with metal ions, such as iron. It can also be generated via water radiolysis.	Neutralized by antioxidants such as glutathione, ascorbate, and mannitol.
H_2_O_2_ Hydrogen peroxide	This is induced by the dismutation of superoxide and the action of some oxidases.	It is primarily eliminated by the action of enzymes, such as catalase, peroxidases, and glutathione peroxidase.
^1^O_2_ Singlet oxygen	Generated from triplet molecular oxygen through photoexcitation or photochemical reactions.	Deactivated by glutathione, carotenoids, and tocopherols, which act as energy quenchers.
Reactive Nitrogen Species (RNS)		
NO^∙^ Nitric Oxide	Enzymatically synthesized by nitric oxide synthase (NOS).	It reacts rapidly with oxygen, superoxide, and hemoglobin to form other nitrogen compounds.
ONOO^−^ Peroxynitrite	This was induced by the reaction of nitric oxide with superoxide radicals.	It is eliminated by the action of glutathione peroxidase, peroxiredoxins, or by spontaneous decomposition.
NO_2_^∙^ Nitrogen dioxide	It is generated by the oxidation of nitric oxide or peroxynitrite.	Reacts with antioxidants (glutathione, ascorbate) and water to form nitrite and nitrate.

**Table 2:** Effect of pharmaceutical products on biomarkers of oxidative stress in amphibians.

Specie	Pharmaceutical	Sample Size and Stage	Concentration (μgL^−1^)	Time of Exposure	Methodology	Main Findings	References
*Limnodynastes peronii*	Diclofenac Naproxen Atenolol Gemfibrozil	50 full operculum-stage larvae (Gosner stage 25) were used for each treatment.	0.1, 1, 10, and 100	30 days	All biomarkers were analyzed using a FLUOstar Omega plate reader.	A significant increase in peroxidase activity was observed at the highest concentration of the drug mixture.	[69]
*Pelophylax ridibundus*	Nifedipine	40 adult male frogs were utilized for oxidative stress experimentation.	3463.4	14 days	Absorbance was measured with a UV/Vis spectrophotometer “LOMO-56” and fluorescence was measured with the f-max fluorescence microplate reader.	Increased ROS production, elevated SOD activity, and higher GSH and GSSG levels.	[70]
*Pelophylax ridibundus*	Ibuprofen Estrone	15 adult male frogs per experimental group were used.	0.25 0.1	14 days	The absorbance was measured on the UV/Vis spectrophotometer “LOMO-56” (LOMO, Russian Federation), and the fluorescence was measured on the f-max fluorescence microplate reader.	Exposure can induce oxidative stress, although the magnitude of this effect varies depending on the compound.	[71]
*Rhinella arenarum*	Enrofloxacin Ciprofloxacin	For the oxidative stress tests, 10 larvae per treatment at Gosner stages 28–29 were used.	1, 10, 100, and 1000	96 h	Spectrophotometric methods.	An increase in LPO, decrease in CAT activity, and increase in GST activity was observed, particularly at the highest exposure concentrations.	[72]
*Trachycephalus typhonius* *Physalaemus albonotatus*	Diclofenac	10 larvae per treatment and control per species were used.	125 to 4000 125 to 2000	96 h 22 and 20 days	Using a Jenway 6405 UV/Vis spectrophotometer.	An imbalance between ROS production and antioxidant systems was observed in both species, whereas GST activity exhibited interspecies variation.	[73]
*Rhinella arenarum*	Dexamethasone	10 surviving larvae (Gosner stage 38) from each dexamethasone treatment and control were used.	1–1000	22 days	Spectrophotometrically using a JENWAY UV/Vis spectrophotometer.	GST activity significantly increased in larvae exposed to the drug.	[74]
*Rhinella arenarum*	Lamivudine Stavudine Zidovudine Nevirapine	15 tadpoles per treatment were utilized at Gosner stage 26–28.	500, 1000, 2000, and 4000	48 h	A Jenway 6405 UV/Vis spectrophotometer was utilized to evaluate enzymatic activities	Biochemical imbalance between ROS production and induction of antioxidant systems.	[75]
*Physalaemus cuvieri*	Hydroxychloroquine Azithromycin	96 tadpoles at Gosner stage 26 were used per group.	12.5	72 h	Absorbance was measured with a UV/Vis spectrophotometer and fluorescence was measured with a f-max fluorescence microplate reader.	Exposure to drugs did not elicit a significant oxidative stress response in tadpoles, potentially because of the activity of antioxidant enzymes.	[76]
*Xenopus laevis*	Delorazepam	In total, 360 embryos were used in this study. The embryos were exposed starting at the 4/8 cell stage.	1, 5 and 10	96 h	The absorbance for HPs, GPX, and GR was measured using a multi-mode microplate reader (Synergy™ HTX Multi-Mode Microplate Reader, BioTek).	Delorazepam alters redox equilibrium in embryos, potentially resulting in adverse effects on their development and viability.	[77]
*Rhinella arenarum*	Oxytetracycline	A cohort of 50 embryos (stage 4) and an equal number of larvae (stage 25) were exposed to sublethal concentrations of OTC.	1, 3, and 6 × 10^7^	96 h	All biochemical determinations were performed in duplicate using a Perkin Elmer UV/Vis Lambda 35 spectrometer.	Exposure induced oxidative stress in both embryos and larvae, as evidenced by increased lipoperoxidation and altered antioxidant enzyme activities.	[78]
*Lithobates catesbeianus*	Sulfamethoxazole Oxytetracycline	This investigation employed 160 tadpoles, specifically those in developmental stages 32–36.	0.02, 0.09, and 0.46	16 days	Oxidative stress biomarkers were determined using spectrophotometric and high-performance liquid chromatography (HPLC) techniques.	Drug exposure induced OS in tadpoles as evidenced by the inhibition of antioxidant enzymes and increased oxidative damage to proteins.	[79]
*Hyla arborea*	Ethyl 3-aminobenzoate methanesulfonate (MS-222)	A total of 96 tadpoles at Gosner stage 40 were used to assess the effects of MS-222 on oxidative status parameters.	1 × 10^5^, 1, and 5 × 10^6^	15 min	A UV/Vis spectrophotometer (UV-1800, Shimadzu, Japan) was used to determine antioxidant parameters, and a plate reader (Synergy H1, BioTek Instruments, Winooski, VT, USA) was used to measure the levels of LPO and PCO.	MS-222 may potentially interfere with investigations of OS biomarkers, particularly those associated with GSH.	[80]
*Xenopus laevis*	Favipiravir Oseltamivir	A total of 32 embryos at stage 8–11 were used.	3.29 × 10^4^ to 2.5 × 10^5^ 8.2 × 10^3^ to 6.25 × 10^4^	96 h	A spectrophotometer (Shimadzu, UV-1601) was used to determine the catalase (CAT) activity. A microplate reader (VersaMax, Molecular Devices Corp., San Jose, CA, USA) was used to determine the levels of oxidative stress biomarkers GST, GR, CaE, and AChE.	Biomarker responses indicate distinct detoxification and oxidative stress processes during organogenesis and the subsequent developmental stages.	[81]
*Rhinella arenarum*	Ivermectin	50 embryos (stages 4–6, blastula to gastrula) or 50 larvae (stage 25, complete operculum) were used.	1.25, 10 and 100	96 h	A plate reader (Synergy H1, BioTek Instruments, USA) was used to measure oxidative stress biomarkers.	Induced OS, even at low concentrations, and the commercial formulation may exhibit higher toxicity than the active ingredient alone.	[82]
*Rhinella arenarum*	Monensin	50 embryos at stage 4 (blastula) and 50 larvae at stage 25 (complete operculum) were used to evaluate the effect of monensin on oxidative stress biomarkers.	4, 12 and 120	96 h	A spectrophotometer (Perkin Elmer UV/Vis Lambda 35) was used to determine CAT, GST, GSH, and TBARS levels.	A decrease in GST activity and GSH levels was observed, which was accompanied by an increase in TBARS levels.	[83]
*Aquarana catesbeianus*	Prednisone Prednisolone	A total of 16 tadpoles at Gosner stage 26–27 were used to assess the effects on oxidative status parameters.	0.1, 1 and 10	16 days	A spectrophotometer (SHIMADZU 1650 PC) and microplate reader (FLUOstar Omega BMG LABTECH) were used to determine the oxidative stress biomarkers.	Elevated SOD, CAT, GPx, and GST activities as well as increased MDA levels were observed in tadpoles exposed to prednisone.	[84]
